# Implementing epic fast pass for echocardiogram and endoscopy to improve healthcare access and utilization

**DOI:** 10.1038/s44401-024-00005-0

**Published:** 2024-12-23

**Authors:** Andrew L. L. Yin, Andrew M. Feigelman, Yahaira Delgado, Robert J. Min, Adam D. Cheriff, J. Travis Gossey, Richard Trepp, Ashley Beecy

**Affiliations:** 1 Weill Cornell Medicine, New York, NY USA; 2 NewYork-Presbyterian, New York, NY USA; 3 Columbia University Irving Medical Center, New York, NY USA

**Keywords:** Information systems and information technology, Operational research, Health care, Medical imaging

## Abstract

Access and efficient resource utilization remain critical challenges in healthcare, often leading to long wait times despite unfilled outpatient appointments. Epic Fast Pass (EFP), an innovative feature within the Electronic Health Record (EHR), has improved outpatient appointment scheduling, reduced wait times, and enhanced access in routine appointments. Guided by the principles of a learning health system, we describe the novel application of EFP to imaging and procedural services, specifically outpatient echocardiograms and endoscopies. We collected user data over 15 months for echocardiograms and 4 months for endoscopies. For echocardiograms, 41.26% of patients accepted an offer, improving their appointment times by an average of 12.8 days. For endoscopies, 48.35% accepted, with an average improvement of 50.43 days. Our results demonstrate that rescheduling tools for outpatient imaging and procedural appointments are both feasible and promising, with the potential to enhance patient access and optimize resource utilization.

## Introduction

This study reports the design and early implementation of Epic Fast Pass (EFP), an existing tool in Epic, an Electronic Health Record (EHR), for the automated rescheduling of outpatient procedural appointments, specifically for echocardiograms and endoscopy in a large academic medical center in a busy metropolitan area. Here, we describe the design, organizational process, and report early findings along with early implementation data from the initial roll out of the new system in accordance with recent aims for standardization in digital health implementations^1,2^,

Appointment scheduling and access remains a key challenge when it comes to healthcare utilization and patient outcomes. No-shows, overbooking, and long wait times all play an important role in healthcare expenses, patient outcomes, and patient experience^3–7^. Maximizing utilization of outpatient resources is often time consuming and administratively burdensome, involving clinic staff actively identifying and calling patients to offer earlier appointments, calling to remind patients, or other manual and labor-intensive processes^8,9^. Identifying the right patient entails understanding of insurance (prior authorization), resource capability (room or machinery), and staffing (personnel to perform the procedure)^4,10^. Our work aims to address the health system challenges of availability, quality, and cost as described by the WHO Classification of Digital Health Interventions^11^.

Patient portals have been shown to be beneficial to clinical outcomes such as medication adherence and preventative services and have had increasing use over the years^12–14^. EFP is a standard tool within Epic for automated rescheduling of outpatient office visits. Prior work has shown that patients who reschedule through EFP are more likely to complete their appointment and also less likely to cancel or no-show their appointment. Even patients who simply viewed offers were more likely to show up or cancel their original appointment time^4,15^. This study presents an added value by demonstrating the feasibility of bringing EFP to the outpatient imaging and procedure related spaces, specifically echocardiogram and endoscopy, which has not been described previously. No-shows and empty appointment slots in these spaces include a cost of not only the provider or technical team that is left unutilized, but also the idling of the machinery or equipment required for the test or procedure. Scheduling in these spaces, however, is often more complicated as they include factors such as prior authorizations for insurance approval, appropriate staffing and resource matching for more specialized resources, or required patient preparation (e.g., colonoscopy prep) that are generally not prevalent in a standard outpatient appointment.

## Results

### Coverage

After a brief pilot, this intervention was released within Weill Cornel Medicine (WCM) for all echocardiograms at the WCM main campus at the beginning of 2023. The coverage of the current intervention is local to the WCM outpatient practice for patients who have MyChart (MyChart® is a registered trademark of Epic Systems Corporation). At the end of 2023, 91.7% of all outpatients at WCM were enrolled in MyChart, including 91.3% of all Cardiology patients and 94.2% of all Gastroenterology patients. For echocardiogram, this intervention included all rooms and transthoracic echocardiograms in the WCM outpatient clinic. After this release in 2023, EFP was released for screening colonoscopies at the beginning of 2024 for all patients referred by their primary care providers for screening colonoscopies who were enrolled in MyChart. This expanded to also include esophagogastroduodenoscopy (EGD) referrals made by primary care providers or those triggered through routine screening questionnaires.

### Outcomes

For echocardiogram, results were collected over the course of 14 months. Over this time period, 143 patients were included. Basic demographics of these patients can be seen in Table 1. 1786 appointment offers were made to patients using EFP. Of those offers, 59 were accepted, 214 offers were declined, 1452 expired, and 61 were no longer available when viewed due to the appointment slot being taken by someone else. Of the accepting patients, 52 showed up for their appointment, 5 cancelled, and 2 did not show up. 1106 offers were viewed by patients. Patients experienced an average of 12.8 days improvement over their original appointment date with a median improvement of 7.5 days and range of 3 to 119 days improvement. Mean age was 58.23 and 59.8 for interactors and non-interactors, respectively with no significant difference between these two groups observed (*p* = 0.65). These results can be seen in Table 2.

**Table 1:** Echocardiogram Demographics

Echocardiogram	Total (*N* = 143), *n*(%)	Interactor (*N* = 98), *n*(%)	Non-Interactor (*N* = 45), *n*(%)	X^2^, *P*-value
Gender				0.018, 0.11
Male	90 (62.94)	32 (32.65)	21 (46.67)	
Female	53 (37.06)	66 (67.35)	24 (53.33)	
Age				2.28, 0.68
< 45	40 (27.97)	27 (27.55)	13 (28.89)	
45–65	41 (28.67)	27 (27.55)	15 (33.33)	
> 65	61 (42.66)	44 (44.90)	17 (37.78)	
Ethnicity				0.011, 0.006
Declined	25 (17.48)	13 (13.27)	12 (26.67)	
Non Hispanic	113 (79.02)	84 (85.71)	29 (64.44)	
Hispanic	5 (3.5)	1 (1.02)	4 (8.89)	
Race				1.62, 0.20
Asian	12 (8.39)	9 (9.18)	3 (6.67)	
Black	12 (8.39)	8 (8.16)	4 (8.89)	
Declined	16 (11.19)	7 (7.14)	9 (20.00)	
Other	10 (6.99)	6 (6.12)	4 (8.89)	
White	93 (65.03)	68 (69.39)	25 (55.56)	

Demographic data for patients included in EFP for echocardiogram. Interactors were defined as those patients who either accepted or declined an offer, while non-interactors were those who did not respond or interact with any given offer.

**Table 2:** Echocardiogram actions

Echocardiogram	
Number of Patients included	143
Total Offers	1786
Accepted offers	59
Declined offers	214
Expired offers	1452
Appt no longer available	61
Average Days improvement for accepted appointments	12.8
Completed appointments	52 (88.14%)
No shows	2 (3.79%)
Cancellations	5 (8.20%)
Offers viewed	1106 (61.93%)
Offers viewed within 1 h of release	240 (13.44%)

This table shows a breakdown of actions and behaviors of patient’s included in EFP for echocardiogram.

For endoscopy, results were collected over the course of 4 months. Over this time period, 666 patients were included. Basic demographics of these patients can be seen in Table 3. Over this time, 9338 appointment offers were made to patients using EFP. Of those offers, 322 were accepted, 821 offers were declined, 7640 expired, and 533 were no longer available when viewed due to the appointment slot being taken by someone else. Of the accepting patients, 168 showed up for their appointment, 105 were currently scheduled, 47 cancelled, and 2 did not show up. 6588 (70.55%) offers were viewed by patients. Patients experienced an average of 50.43 days improvement over their original appointment time with a median improvement of 41 days and a range of 7 to 199 days improvement. For the 217 patients who had passed their appointment time at the time of data collection, the no-show rate was less than 1% and cancellation rate was 21.66%. In terms of type of endoscopy procedure, 95.7% of offers were for rescheduling a colonoscopy and 4.3% EGD. Mean age was 54.39 and 54.60 for interactors and non-interactors, respectively, with no significant difference between these two groups observed (*p* = 0.82). These results can be seen in Table 4.

**Table 3:** Endoscopy Demographics

Endoscopy	Total (*N* = 666), *n*(%)	Interactor (*N* = 443), *n*(%)	Non-Interactor (*N* = 223), *n*(%)	X^2^, *P*-value
Gender				0.0026, 0.04
Male	242 (36.34)	149 (33.63)	93 (41.7)	
Female	424 (63.66)	294 (66.37)	130 (58.3)	
Age				1.34, 0.49
< 45	73 (10.96)	44 (9.93)	29 (13.00)	
45–65	477 (71.62)	321 (72.46)	156 (69.96)	
> 65	116 (17.42)	78 (17.61)	38 (17.04)	
Ethnicity				0.184, 0.09
Declined	155 (23.27)	98 (22.12)	57 (25.56)	
Non-Hispanic	457 (68.62)	315 (71.11)	142 (63.68)	
Hispanic	54 (8.11)	30 (6.77)	24 (10.76)	
Race				0.986, 0.09
Asian	51 (7.66)	35 (7.9)	16 (7.17)	
Black	62 (9.31)	38 (8.58)	24 (10.76)	
Declined	125 (18.77)	77 (17.38)	48 (21.52)	
Other	72 (10.81)	41 (9.26)	31 (13.9)	
White	356 (53.45)	252 (56.88)	104 (46.64)	

Demographic data for patients included in EFP for endoscopy. Interactors were defined as those patients who either accepted or declined an offer, while non-interactors were those who did not respond or interact with any given offer.

**Table 4:** Endoscopy actions

Endoscopy	
Number of Patients included	666
Total Offers	9338
Accepted offers	322
Declined offers	821
Expired offers	7640
Deleted Offers	2
Appt no longer available	533
Average Days improvement for accepted appointments	50.43
Completed appointments	168 (77.42%)
No shows	2 (0.92%)
Cancellations	47 (21.66%)
Scheduled	105
Percent offers viewed	6588 (70.55%)
Percent offers viewed within 1 h of release	1311 (14.04%)

This table shows a breakdown of actions and behaviors of patient’s included in EFP for endoscopy.

In echocardiogram, using chi-square to assess difference in demographics, patients who interacted with EFP (by either actively accepting or declining an offer at some point) differed significantly in ethnicity, tending to be more non-Hispanic than those who did not interact (*P* = 0.006). They also tended to be female (*P* = 0.11) and white (*P* = 0.20) although these did not achieve significance. Age did not seem to be a factor (*P* = 0.68).

In endoscopy, using chi-square to assess difference in demographics, patients interacting with EFP were significantly more likely to be female (*P* = 0.04) and were more likely to be non-Hispanic (*P* = 0.09) and white (*P* = 0.09) although these did not reach a significant threshold. Again, age did not seem to be a major factor (*P* = 0.49).

For complete chi-square tables please see supplemental tables.

## Discussion

EFP was successfully deployed into outpatient echocardiogram and endoscopy scheduling. To our knowledge, this is the first time EFP has been used for procedures or imaging exams. Our results showed that the implementation was feasible, easily accepted with low demand on clinic and EHR team resources and minimally problematic for patients to readily use and understand. Given the importance of maximizing resource utilization in healthcare as well as improving patient’s access to care, this intervention has proven to be a simple way to address both of these. Given the known success of EFP for outpatient office visits, lessons learned here are key to the expansion of this service to other settings with the goal of continued success and added value.

Importantly, 41.26% and 48.33% of patients included in echocardiogram and endoscopy accepted an EFP offer, respectively, illustrating that nearly half the patients involved in both interventions were able to move up their appointment. For our primary outcome, patients accepting offers experienced an average of 12.5 days improvement in their echocardiogram and 50.43 days improvement in their endoscopy timing, with a median improvement in endoscopy of 41 days. In terms of all EFP offers sent, this translated to acceptance rates of 3.30% and 3.45% for echocardiogram and endoscopy appointments, respectively. This is lower than the 8% and 11% seen for outpatient appointments that has been seen in other recent studies^4,15^, but it is important to note that acceptance rates using EFP in outpatient scheduling at WCM is lower at baseline at 6.36%. Additionally, no-show rates for echocardiogram and endoscopy for accepted appointments were 3.79% and 0.92%, respectively, which are lower than the no-show rates observed in EFP for outpatient appointments and these procedures when scheduled without using EFP and in line or lower with those seen in prior studies^4,15^. Given the additional complexity and parameters required for rescheduling an imaging exam (prior authorization, resource availability, patient preparation, technical capabilities of the given resource) over a simple office visit, we feel this is a strong starting point for this early intervention and can be used as a playbook when applied to other procedures or imaging studies. Even so, further data and work are necessary to better understand nuances in the results. In alignment with prior studies, patient’s interacting with EFP in both echocardiogram and endoscopy in our cohorts were more likely to be white and non-Hispanic. In contrast to these prior studies, patients tended to be more female and age did not appear to be a significant factor^4,15^. Some of these observations are consistent with known disparities in portal use amongst demographics and also reflect some differences in geographies of different studies, the understanding of which continues to be an area of study, especially since the COVID pandemic^16–19^. Additionally, WCM has not engaged in any systematic marketing or other MyChart engagement campaigns targeting different age groups at WCM to explain why age is not a factor in our study.

We believe this current work exemplifies the value of creating a learning health system and the use of its principles to guide action within a healthcare system. The 6-stages of a rapid-learning health care system provided the general blueprint for our process. An observed backlog in patients waiting for echocardiograms began as our initial internal challenge with the decision to use EFP. This tool had been successfully used in other areas, was already integrated into our medical record system, and had many staff that were familiar with its use. With this, we rapidly piloted, evaluated, and adjusted EFP for novel use in an imaging setting to fit for echocardiograms. In exploring other potential uses for EFP in imaging or procedures, we found that EFP had uses elsewhere in other departments with long waiting lists for patients. In this case, within months we were able to implement EFP to procedural appointments including screening colonoscopies and some endoscopies. As described by Friedman et al., our success in moving from one area to another came from the successful foundations for a learning health system. This included having trusted and valued stakeholders in ITS, our medical record provider, our clinics, and our implementation team as well as an adaptable and responsive system that allowed for sharing of successes across departments and data streams. This is possible in an organization that has a shared incentive to improve access to care for our patients and streamline their experience, allowing staff the space to pursue individual ways to achieve these measures^20,21^. We discuss some specific success factors and challenges below.

A significant advantage was that the EFP was already in use for outpatient office visits. This familiarity allowed all parties to readily grasp the concept and intentions behind extending it to imaging and procedural services, enabling the IT team to develop new logic and templates without needing to create an entirely new tool. Equally critical were choosing services with long wait times and simple scheduling protocols. Our evaluation at WCM showed a wide range of waitlist length and scheduling complexity. The choice of echocardiogram as the initial procedure was pivotal to the success of the project. As a non-invasive procedure that requires no specific patient preparation and utilizes an interchangeable resource, for example, any technician can perform the procedure in any available room, it provided an ideal test case. This success set the stage for expansion to a more complex procedure of endoscopy, which requires additional patient preparation (e.g. colonoscopy or endoscopy preparation) and specific provider and resource allocations (e.g., specific providers have specific procedure time and availability). Moreover, the management of insurance and authorization processes was important. Each office had established protocols for obtaining and confirming prior authorizations from insurance, which streamlined preparations for each procedure. In echocardiogram, the implementation began with Medicare patients for whom no prior authorization was required and after establishing this workflow was then expanded to include all insurance types. With the implementation of EFP, clinic staff reported an increased volume of authorizations that were required given the quicker patient rescheduling and higher volume of rescheduled appointments. By starting in echocardiogram, we were able to learn the basic necessities for each of the above parameters – resource requirements, scheduling needs, insurance and prior authorization protocols – and take them to the endoscopy team as previously developed workflows. With this foundation we were able to customize the intervention further to the specific needs of endoscopy. Although additional work needs to be done in this area, so far, there have been no reported patient complaints regarding the use of EFP. Staff have similar reported no major issues regarding using the waitlist system. Although EFP has been implemented in parallel with other initiatives, the waitlist at the endoscopy clinic has excitingly seen significant improvement since implementation with wait times from roughly 6 months down to just a few weeks.

Alongside the factors that facilitated success, the implementation of EFP faced several challenges. Initially, there was an ambitious attempt to deploy EFP across various imaging modalities simultaneously to optimize resources and streamline changes. Numerous radiology and testing studies were found to have little no wait time, thus EFP had no role to provide benefit. The implementation team also encountered complexities due to the unique parameters and infrastructure dependencies of different procedures. For instance, when moving from echocardiogram to screening colonoscopies, we revealed scheduling logic errors in the medical record referral orders. These had been previously insignificant in affecting patients, but for our implementation led to the offering of incorrect appointments and times. Additional errors included allocating resources—such as rooms or providers—that were unavailable or inappropriate, misassigning patients to the wrong providers, or scheduling unsuitable resources for specific procedures. These challenges highlighted the nuanced understanding each staff member has of the system, including provider preferences and specific exam requirements, which needed to be integrated into EFP’s logic. Building trust with staff became crucial yet difficult, as EFP’s effectiveness depended on accurately managing a waitlist system, staff education and attention to a new workflow, and promptly identifying and rectifying errors. One later challenge in echocardiogram was the change of the WCM call center personnel after the EFP guidelines had been implemented. This led to a significant drop off in adding patients to the waitlist given the new staff was unaware of the EFP protocol, which is required for EFP. This serves as an example of how seemingly innocuous system level changes can have many trickle-down effects on existing systems. Without active monitoring of EFP, this decrease in utilization would possibly have gone unnoticed. Furthermore, integrating this new workflow occasionally proved problematic for teams, particularly in managing the diverse timelines required for prior authorizations for different procedures. This aspect of the implementation required careful handling to accommodate the varied procedural demands efficiently.

As this current implementation continues to build, future work will look to understand more of the user perspective regarding experience with the implementation and understand more about the different demographics that seem to contribute to patients being more or less likely to interact with the system. Future directions will also hope to use the playbook created from echocardiogram and endoscopy implementation to expand this tool into other imaging and procedural appointments. We also acknowledge the limitation in our implementation only including patients who actively have an online portal within the institution. Although the proportion of patients in this population who use the portal is high ( > 90%), it still leaves a portion of patients who are not included in this implementation. It will be important for future work to evaluate clinic-wide effects of the implementations and better analyze the metrics for the clinic outcomes and the effects on staffing needs.

In this work, we describe the implementation of EFP into the workflow of echocardiogram and endoscopy appointment scheduling. To our knowledge, we are the first to demonstrate that EFP is a tool that can be successfully used in imaging or procedural appointments, with a detailed description of the process of our implementation and the results. Our findings indicate the EFP is able to be implemented into imaging and procedural appointment scheduling and provides optimism about expanding EFP into other imaging and procedural appointment scheduling processes in order to streamline scheduling for staff and patients as well as improve utility of healthcare resources.

## Methods

### Aims and objectives

Our objective was to develop and deploy a system that enhances the utilization of procedural and imaging modalities at a large, academic medical institution. We delved into patient interactions with EFP, with the primary outcome of days of improvement over the original appointment time. We analyzed behavior metrics such as the time taken to view and respond to offers, and the number of offers received. Additionally, we identified potential barriers that could hinder the broad application of EFP to other procedures or health systems. This involves both quantitative monitoring of appointment outcomes, including lead times and no-show rates, and qualitative assessments through discussions with office staff regarding scheduling errors or inefficiencies. We accessed biases that may be present in the system. We hope to showcased the effectiveness of adopting a learning health system model in creating and refining these implementations, demonstrating clear benefits and promoting continuous improvement in healthcare delivery.

### Blueprint summary

Following the successful application of EFP to outpatient office visits and the notable increase in patient engagement with digital health platforms post-COVID-19 pandemic, it was fitting to extend EFP’s utility to procedural and imaging services. A collaborative team consisting of clinicians, administrative staff, information technologists, and Epic analysts from our organization assembled to design a workflow tailored for this application. We chose echocardiograms as our initial focus due to their non-invasive nature, lack of patient preparation requirements, and extensive wait times seen in our organization. All patients for whom an echocardiogram was ordered by their provider were placed on the waitlist to be included in EFP.

Building on our experiences with echocardiograms, we next targeted outpatient screening colonoscopies, motivated by similar issues of prolonged wait times and high demand. In our health system, primary care providers can refer patients for a routine screening colonoscopy. These patients do not need to see a gastroenterologist prior to colonoscopy and can schedule with any provider available. Office staff call these patients to confirm an initial appointment time and then those with active myChart who need an earlier appointment than what is currently available were added to the EFP waitlist. We then expanded this effort to include outpatient esophagogastroduodenoscopy (EGD) referrals generated through referrals from primary care providers or screening questionnaires that were positive for evidence of upper gastrointestinal symptoms and indicating the need for EGD evaluation. These patients see a provider before being scheduled and were added to the EFP waitlist if the soonest available time was longer than that recommended by the clinical evaluation.

Through these initiatives, we tackled several pressing issues outlined by the World Health Organization’s Health System Challenges, including inadequate equipment supply, need to improve patient experience, inefficient resource allocation, and the high costs associated with manual processes^11^. We are committed to enhancing healthcare delivery and patient satisfaction across the board. Our approach in this intervention aims to align with the merits of a learning health system that is able to combine technology, data, and process to improve health. We describe our intervention’s ability to match to the 6 phase rapid-learning health system as has been described previously^20,21^.

### Technical design

**Fig. 1:**
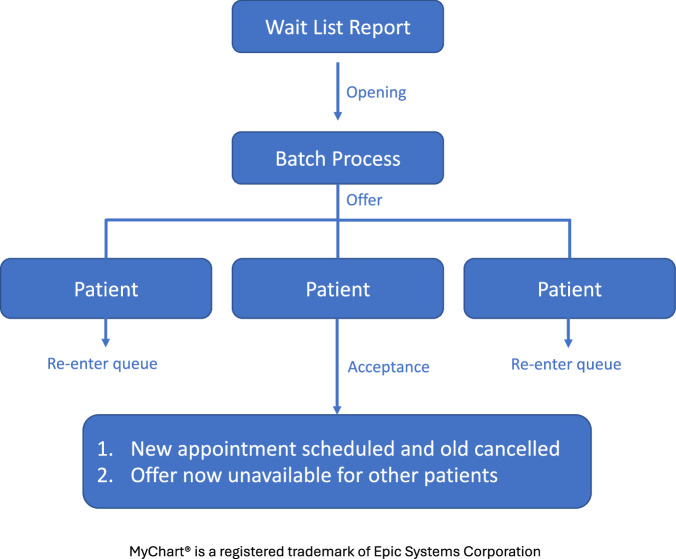
EFP flow. As adapted from the Epic User manual, this displays the basic flow for the EFP system for how patients receive offers for appointments. The batch process can be customized to determine which openings and which patients should be selected to receive offers.

EFP is a tool within the EHR that facilitates filling empty appointment slots by offering them to patients with active portal accounts who have been entered on a waitlist. Staff add patients with scheduled appointments to the waitlist in the EHR for a given appointment type, procedure, or provider. Patients are then offered newly empty appointment slots through their MyChart with the option to accept or decline an offered timeslot. Patients can respond to these offers either through MyChart or by responding to a text message they receive. Initial team meetings focused on the value of the intervention including the potential for time saved by clinic staff as well as reduction of wait times for patient appointments. Discussions also explored selecting the appropriate procedure or imaging exam for the intervention. Procedures and imaging exams often require prior authorizations, which the office confirms is complete prior to the visit. The timeframe needed to complete the prior authorization determined how much lead time was needed for any rescheduled appointment. An overview of the appointment offering workflow, adapted from the Epic user manual, can be seen in Fig. 1.

### Target

**Fig. 2:**
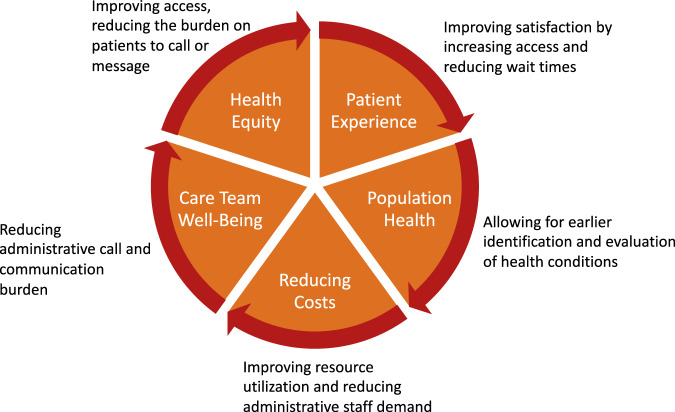
Quintuple aims. Quintuple aim of healthcare improvement along with how the current intervention aims to address each of the aims.

Although EFP most clearly targets the patients as the main user of the tool, equal targets in this intervention were the clinic staff normally responsible for filling empty appointments and also the staff who make the rescheduling logic and resulting adjustments to certain workflows. This involves the administration team needed to add patients to the waitlist, the clinical outpatient office managers responsible for resource management, and the Epic analysts from our institution needed to complete the design and build. All together, we chose this intervention with the belief that it could potentially address all components of the quintuple aims of healthcare improvement – patient experience, population health, reducing costs, care team well-being, and health equity as illustrated in Fig. 2^22^.

### Data

Data relating to the intervention is available in Epic Reports on EFP, a pre-existing data synthesis tool within Epic. As EFP is a component of Epic, all offers are recorded in the system and patient responses are also recorded with the same system. Data abstracted from Epic Reports for analytic purposes were stored on REDCap, a free data management software system for the creation of customized, secure data management systems^23^. Descriptive statistics were performed. We compared differences between “interactors” (those who actively accepted or declined an offer) and “non-interactors” (those who did not interact with the system) using the chi-square test. We also performed paired two tailed two sample t-testing of unequal variances between the age of interactors and non-interactors to evaluate if age was a significantly contributor. Office managers and the implementation team monitor the status of waitlists, as these are managed in Epic. Individual patient data were more closely queried for troubleshooting and error analysis on an as needed individual basis. The Weill Cornell Medicine (WCM) Institutional Review Board approved this study and data governance structure (#23-04025928). A waiver of consent was obtained.

### Participating entities

Weill Cornell Medicine (WCM) employs approximately 1600 multi-specialty attending physicians delivering both inpatient and outpatient care in collaboration with NewYork-Presbyterian Hospital. The outpatient echo department has 8 echo rooms and performed 21,763 echocardiograms in 2023. This department serves to provide echocardiograms to both the cardiology department of WCM as well as all other departments that refer patients for echocardiograms. The outpatient endoscopy department performed 16,340 colonoscopies and 10,835 upper endoscopies in 2023. Key partners included the physician organization and informatics leadership, IT team, the clinic office staff, and the implementation team itself. There were no significant funders involved in the study.

### Cost and sustainability

The costs associated with this project were low, with the primary expense being the time invested by the clinical operations and IT teams, as well as the implementation team. These groups conducted a series of meetings to review the existing system, designed a tailored build for the new implementation, and engaged in follow-up sessions to gather feedback and make necessary adjustments. Additionally, clinical operations devoted extra time to train staff on the new workflow. In total, the project involved approximately two months of weekly meetings involving all or selected members of the groups involved. Once deployed, there is minimal to no cost associated with maintenance. Occasional communication between department administrators and office staff is needed to reinforce maintenance of the waitlist or correct for EHR updates. Once deployed, the system requires no more maintenance than common EHR support for all issues.

## Supplementary information

EFP Supplemental Tables

## Data Availability

The data that support the findings of this study are not openly available due to reasons of sensitivity and are available from the corresponding author upon reasonable request.
